# Urinary fluoride levels and metal co-exposures among pregnant women in Los Angeles, California

**DOI:** 10.1186/s12940-023-01026-2

**Published:** 2023-10-26

**Authors:** Ashley J. Malin, Howard Hu, E. Angeles Martínez-Mier, Sandrah P. Eckel, Shohreh F. Farzan, Caitlin G. Howe, William Funk, John D. Meeker, Rima Habre, Theresa M. Bastain, Carrie V. Breton

**Affiliations:** 1 Department of Epidemiology, College of Public Health and Health Professions and College of Medicine, University of Florida, 2004 Mowry Rd, Gainesville, 32603 USA; 2 Department of Population and Public Health Sciences, Keck School of Medicine of University of Southern California, 1845 N Soto Street, Los Angeles, CA 90089-9239 USA; 3 Department of Cariology, Operative Dentistry and Dental Public Health, School of Dentistry, Indiana University, 1121 W. Michigan St., Indianapolis, IN 46202-2876 USA; 4 Department of Epidemiology, Geisel School of Medicine at Dartmouth, 1 Medical Center Drive, Williamson Translational Research Building, 7th Floor, Lebanon, NH 03756 USA; 5 Department of Preventive Medicine, Feinberg School of Medicine, Northwestern University, 680 N Lake Shore Dr Ste 1400, Chicago, IL 60611 USA; 6 Department of Environmental Health Sciences, University of Michigan School of Public Health, 1420 Washington Hts, Ann Arbor, MI 48109 USA

**Keywords:** Fluoride, Metals, Pregnancy, Biomarkers, Hispanic Women, Los Angeles

## Abstract

**Background:**

Fluoride is ubiquitous in the United States (US); however, data on biomarkers and patterns of fluoride exposure among US pregnant women are scarce. We examined specific gravity adjusted maternal urinary fluoride (MUFsg) in relation to sociodemographic variables and metal co-exposures among pregnant women in Los Angeles, California.

**Methods:**

Participants were from the Maternal and Developmental Risks from Environmental and Social Stressors (MADRES) cohort. There were 293 and 490 women with MUFsg measured during first and third trimesters, respectively. An intra-class correlation coefficient examined consistency of MUFsg between trimesters. Kruskal–Wallis and Mann-Whitney U tests examined associations of MUFsg with sociodemographic variables. Covariate adjusted linear regression examined associations of MUFsg with blood metals and specific gravity adjusted urine metals among a subsample of participants within and between trimesters. A False Discovery Rate (FDR) correction accounted for multiple comparisons.

**Results:**

Median (IQR) MUFsg was 0.65 (0.5) mg/L and 0.8 (0.59) mg/L, during trimesters one and three respectively. During both trimesters, MUFsg was higher among older participants, those with higher income, and White, non-Hispanic participants than Hispanic participants. MUFsg was also higher for White, non-Hispanic participants than for Black, non-Hispanic participants in trimester three, and for those with graduate training in trimester one. MUFsg was negatively associated with blood mercury in trimester one and positively associated with blood lead in trimester three. MUFsg was positively associated with various urinary metals, including antimony, barium, cadmium, cobalt, copper, lead, nickel, tin, and zinc in trimesters one and/or three.

**Conclusions:**

MUFsg levels observed were comparable to those found in pregnant women in Mexico and Canada that have been associated with poorer neurodevelopmental outcomes. Lower urinary fluoride levels among Hispanic and non-Hispanic Black participants in MADRES compared to non-Hispanic White participants may reflect lower tap water consumption or lower fluoride exposure from other sources. Additional research is needed to examine whether MUFsg levels observed among pregnant women in the US are associated with neurodevelopmental outcomes.

**Supplementary Information:**

The online version contains supplementary material available at 10.1186/s12940-023-01026-2.

## Introduction

Fluoride is widely utilized in North America as a public health intervention for dental caries prevention. The United States (US) is one of the most fluoridated countries in the world with approximately 73% of the population on public water distribution systems receiving fluoridated water [1]. In Los Angeles (LA) County, 89% of cities are at least partially fluoridated [2]; however, the practice became widespread only recently, in 2007 [3]. The US Public Health Service and Health Canada consider a concentration of 0.7 mg/L to be optimal for preventing dental carries, while minimizing risk of adverse systemic health effects [4, 5]. Other countries also utilize fluoride for caries prevention. For example, in Canada, fluoride is added to the public drinking water of approximately 39% of the population [6]. In Mexico, fluoride is added to salt in regions where water fluoride levels fall below 0.7 mg/L [7]. Conversely, most European countries do not have community water fluoridation programs; however, Germany and Switzerland add fluoride to most salt [8]. Still, the World Health Organization considers the optimal concentration for fluoride in drinking water to range from 0.5–1.0 mg/L [9]. Other sources of fluoride for the North American population can include dental products, foods sprayed with fluoride containing pesticides, certain pharmaceuticals, green and black tea, seafood, and food packaging [10–12].

Studies conducted in Mexico and Canada suggest that prenatal fluoride exposure, at levels relevant to the US, may contribute to poorer neurodevelopmental outcomes in offspring, including lower IQ and increased risk of Attention-Deficit/Hyperactivity Disorder [13–17]. While urinary fluoride levels among pregnant women in Canada and Mexico have been characterized [18–20], data on biomarkers and patterns of fluoride exposure among US pregnant women are scarce [21]. Examining patterns of fluoride exposure during pregnancy is important for ultimately assessing whether fluoride exposures at levels that the US population is exposed to may pose risk to the developing fetus. Moreover, since co-exposure to fluoride and toxic metals and/or essential elements can occur, having a better understanding of which metals may interact with fluoride to impact health is also important [22, 23]. Therefore, the current study examined urinary fluoride levels according to sociodemographic factors and metal co-exposures among a cohort of pregnant women residing in urban LA, California.

## Methods

### Participants

The sample consisted of women from the Maternal and Developmental Risks from Environmental and Social Stressors (MADRES) prospective pregnancy cohort. MADRES is an ongoing NIH-funded cohort consisting of over 1000 predominately low-income Hispanic women residing in urban LA. However, not all have provided data. A detailed overview of participant recruitment and data collection for MADRES is described elsewhere [24]. Briefly, pregnant women were recruited beginning in 2015, from prenatal care providers in LA serving predominantly medically underserved communities. Eligibility criteria include being < 30 weeks gestation at the time of recruitment, being ≥ 18 years of age, and speaking English or Spanish fluently. Exclusion criteria include being HIV positive; having a physical, mental, or cognitive disability that would prevent participation or the ability to provide informed consent; current incarceration; and having a multiple gestation pregnancy [24]. The current study includes data from 491 pregnant women in MADRES who had urine collected during the first, third or both trimesters of pregnancy. See Table S1 for a comparison of demographic characteristics between the study sample and all MADRES participants with available demographic data and see Figure S1 for a participant selection flow diagram.

### Sociodemographic variables

#### Pre-pregnancy Body Mass Index (BMI)

Self-reported pre-pregnancy weight was ascertained through interviewer-administered questionnaires during pregnancy. If missing, then the first weight of the index pregnancy (obtained from maternal electronic medical records) was used in lieu of self-reported pre-pregnancy weight. Pre-pregnancy BMI, defined as [weight (kg) / height (cm)^2^] × 10,000, was measured both continuously and categorically. Categorical BMI was initially classified according to the CDC categories of “underweight”, “normal”, “overweight”, “class 1 obese”, “class 2 obese” and “class 3 obese”; however, for the current study, the 3 classes of obesity were collapsed into a single “obese” category. Therefore, the BMI variable was subsequently recoded into a 4-level variable of “underweight”, “normal”, “overweight” and “obese”.

#### Race/ethnicity

Self-reported race and ethnicity were collected separately but were combined for this analysis into a four-category race/ethnicity variable: White non-Hispanic, Black non-Hispanic, Hispanic, and Multiracial or other non-Hispanic.

#### Maternal ethnicity by nativity

Participants were classified according to their self-reported ethnicity and nativity; non-Hispanic, US-born Hispanic, and non-US-born Hispanic.

#### Maternal education

Participants were asked, “what was the last grade in school you completed?” Their responses were classified as less than 12th grade (i.e., did not finish high school), completed grade 12 (i.e., high school), some college or technical school, completed 4 years of college, and some graduate training after college.

#### Smoking history

Participants were asked during at each trimester: “Excluding e-cigarettes, have you ever smoked cigarettes, cigars, or pipes?”. Their responses were coded as “yes” or “no”. A collapsed variable of any smoking during pregnancy and no smoking during pregnancy was then calculated.

#### Earliest ascertained income

Participants were asked during pregnancy, “in which of the following categories did your total household family income fall in the last year?” The categories included, don’t know, less than $15,000, $15,000 to $29,999, $30,000 to $49, 999, $50,000 to $99,999, and $100, 000 or more.

#### Birth order

Mothers were asked what the birth order index of their child was at the time of pregnancy. Birth order was defined as first born, second born, third born, fourth-born, fifth born, sixth-born or later; however, this variable was collapsed into first born, second born and third born or later.

### Urinary fluoride

Single spot urine samples were collected from MADRES participants in 90-mL sterile specimen containers during their first and third trimesters of pregnancy. Women were asked to fast, if possible, prior to attending the study visit. During trimester one, only 46 women reported fasting, while in trimester three most participants (*N* = 385) reported fasting for at least 8 h. Urine samples were transported on ice to the laboratory within 1 h of collection. They were then aliquoted and stored at − 80°C in 1.5 mL sterile cryovials (VWR).

Urinary fluoride was measured at the Oral Health Research Institute, Indiana University, School of Dentistry. Fluoride content of urine samples was quantified using Martinez Mier et al. (2011) modification of the hexamethyldisiloxane (HMDS) microdiffusion method of Taves (1968). Each sample was dispensed into the bottom of a disposable 60 × 15 mm Petri dish and 2.0 mL of deionized water (diH20) was pipetted into each dish. After applying petroleum jelly to the inside of each Petri dish lid corresponding to each sample, 50 WI of 0.05N sodium hydroxide (NaOH) solution was placed in five equal drops on each dish lid. Each dish was then immediately tightly sealed. After burning a small hole into each lid with a soldering iron, 1.0 mL of HMDS-saturated 3N I-12S04 was pipetted in each hole and sealed immediately with petroleum jelly. During overnight diffusion at ambient temperature, fluoride was released and trapped in the NaOH. The trap was recovered and buffered to pH 5.2 with 25 WI of 0.1 M of acetic acid. The recovered solution was adjusted to a final volume of 100 pl with diH20. A similarly prepared standard fluoride curve was used to determine the fluoride content of each sample. Analyses of all standards/samples were performed using a fluoride ion-specific electrode and a pH/lSE meter. Testing included a standard check (using a fluoride standard traceable to NIST) performed with daily sample analysis.

All urine fluoride measurements were adjusted for specific gravity. Urinary specific gravity was measured using a zero-setting calibrated ATAGO@ Pen Refractometer under darkened conditions and was performed daily while setting up Petri dishes for fluoride analysis. Urine fluoride adjusted for specific gravity was derived from the unadjusted fluoride value and specific gravity of each sample using the Levine Fahy equation: [Concentration_SG normalized_ = Concentration_specimen_ (SG_reference_ – 1)/(SG_specimen_ – 1)] where SG_reference_ is the median SG for the cohort [25]. After fluoride analyses was complete, data were reviewed and approved by quality assurance staff and the study director. Out of the 491 participants who had urine collected, all but one who had urine fluoride measured in trimester one also had urine fluoride measured in trimester three (*n* = 293 for urinary fluoride measured in trimester one; *n* = 490 for urinary fluoride measured in trimester three).

### Urine metals

A detailed description of urinary metals measurement has been provided elsewhere [26, 27]. Briefly, urinary metals analyses were performed by NSF International in collaboration with the University of Michigan Children’s Health Exposure Analysis Resource (CHEAR) Laboratory Hub. Metals were measured in urine collected during trimesters one and three using inductively coupled plasma mass spectrometry (ICP-MS). Included in this panel were: antimony (Sb), arsenic (As), barium (Ba), beryllium (Be), cadmium (Cd), cesium (Cs), cobalt (Co), copper (Cu), chromium (Cr), mercury (Hg), manganese (Mn), molybdenum (Mo), nickel (Ni), lead (Pb), platinum (Pt), tin (Sn), thallium (Tl), tungsten (W), uranium (U), vanadium (V), and zinc (Zn). Specific gravity was also measured during the time of urinary metals analyses, and we adjusted urine metals for specific gravity using the Levine Fahy equation described above [25]. Metals with concentrations below the lower limit of detection (LLOD) or in the undetectable range were imputed as LLOD/√2. There were four metals for which >  = 80% of the sample was below the LLOD (i.e., Be, Cr, Pt, and V), and two metals for which >  = 60% of the sample was below the LLOD (i.e., W and U), and therefore they were excluded from statistical analyses. All remaining metals for regression analyses of first trimester MUFsg and first trimester urine metals had < 30% of the sample below the LLOD except for Sb which had 33% below the LLOD. For regression analyses of third trimester MUFsg with third trimester urine metals, all urine metals had < 30% of the sample below the LLOD except for Cd which had 37% below the LLOD and Sb which had 49% below the LLOD.

### Blood metals

Venous whole blood samples were collected from participants during the first and third trimesters of pregnancy during the same visit that the urine samples were collected. Blood metals were measured among a small subset of participants who also had urinary fluoride measured (*n* = 123 in trimester one and *n* = 90 in trimester three). Participants were asked to fast prior to attending the study visit when blood was collected; however, not all were able to. In trimester one, only 34 participants reported fasting for at least 8 hours, while in trimester three most participants with blood metals measured (*n* = 81) reported fasting. Collection time was not standardized. Using acid washed pipette tips, 50 μL of venous whole blood was spiked directly into 15 mL metal-free polypropylene centrifuge tubes (VWR, Atlanta, GA) and extracted in 1.5 mL of 5% ultrapure grade acetic acid and 0.01% ultrapure grade Triton X-100 (Fisher Scientific, Pittsburgh, PA) in 18.2 mΩ deionized water. Two hundred ppb of Au was added to amalgamate Hg to prevent analyte loss throughout the procedure (Inorganic Ventures, Christiansburg, VA). Five ppb of indium, bismuth, and yittrium were added to the extraction solution as internal standards (Inorganic Ventures, Christiansburg, VA). The blood extracts were then centrifuged at 3600 × g for 2 min and incubated for 90 min at room temperature on a shaker table at 300 rpm. As, Cd, Pb, and Hg were quantified in whole blood samples using ICP-MS, performed at the Quantitative Bio-element Imaging Center (QBIC) at Northwestern University. The following isotopes were quantified: ^206^Pd, ^207^Pb, ^208^Pb, ^202^Hg, ^75^As, and ^114^Cd, using previously developed methods [28, 29]. Collisional cell technology was used to eliminate interfering ions. All sample results were above the LLOD.

### Statistical analysis

Statistical analyses were performed using IBM SPSS Statistics version 28. Descriptive statistics were calculated for fluoride, sociodemographic variables, and metals. Specific gravity adjusted maternal urinary fluoride (MUFsg) distributions were skewed, and therefore medians, standard errors, and interquartile ranges are reported. However, we also report arithmetic means and standard deviations for comparison with other fluoride studies. Spearman correlations examined associations of fluoride variables within and between trimesters, and an intra-class correlation coefficient examined consistency of MUFsg between trimesters. Kruskal–Wallis, Mann–Whitney U tests, and Spearman correlations examined associations of MUFsg with sociodemographic variables. Linear regression examined associations of MUFsg with blood and/or urinary metals, adjusted for maternal age, income, pre-pregnancy BMI, ethnicity by nativity and parity, within and between trimesters. We dummy coded covariates including income, parity, and ethnicity by nativity for regression analyses. Additionally, participants with missing data were designated to a “missing” category for these dummy coded covariates. Covariates were selected a prior based on previously established associations between fluoride and metal exposures/ metabolism [18, 30–32]. One participant with an extreme and atypical value of MUFsg = 7.99 during trimester three was removed for all analyses that included third trimester urine. A logarithm base 10 transformation was applied to blood and urine metals to satisfy linear regression assumptions. We conducted sensitivity analyses examining covariate-adjusted associations of MUFsg with blood and/or urinary metals among participants who reported fasting for at least 8 h during trimester 3. A False Discovery Rate (FDR) correction accounted for multiple comparisons for associations between MUFsg and sociodemographic variables for statistically significant Kruskal–Wallis tests, as well as for 60 tests of associations between MUFsg and urinary metals and 16 tests of associations between MUFsg and blood metals within and between trimesters. The criterion for statistical significance was a two-tailed *p-*value or *q*-value of 0.05, depending on the analysis.

## Results

**Fig. 1:**
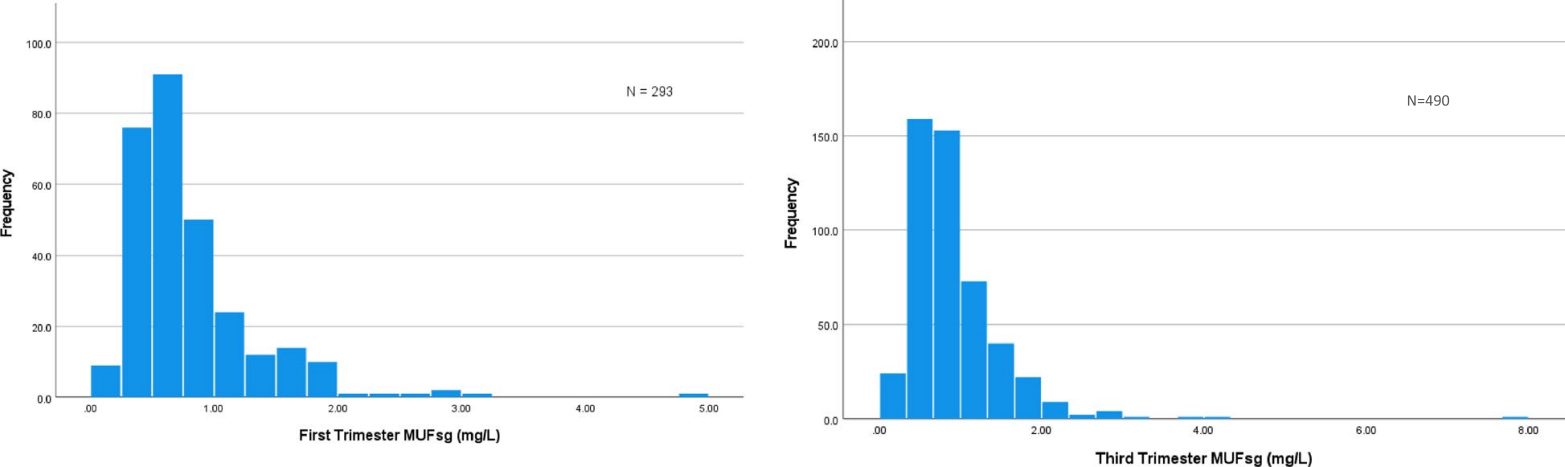
Distribution of MUFsg during the first and third trimesters of pregnancy in the MADRES pregnancy cohort MUFsg = specific gravity adjusted maternal urinary fluoride

Sociodemographic characteristics are presented in Table 1, and descriptive statistics for fluoride measures are presented in Table 2. The mean age of participants was 29 years, and approximately 80% of participants identified as Hispanic or Latina. Most participants had pre-pregnancy BMIs in the overweight or obese categories. The median MUFsg concentration was higher during the third trimester compared with the first trimester (medians = 0.80 mg/L and 0.65 mg/L for trimesters three and one respectively). Distributions of MUFsg during the first and third trimesters are presented in Fig. 1.

**Table 1:** Maternal demographics according to fluoride sample

	**First Trimester Sample (*N* = 293)**	**Third trimester sample (*N* = 490)**
**Age at Consent** (yrs; M, SE)	28.73 (0.34)	28.88 (0.27)
**Pre-pregnancy BMI** (freq., %)		
Underweight	9 (3.1)	13 (2.7)
Normal	84 (28.7)	146 (29.8)
Overweight	93 (31.7)	154 (31.4)
Obese	107 (36.6)	177 (36.2)
**Race** (freq., %) ^a^		
White, non-Hispanic	19 (6.5)	30 (6.2)
Black, non-Hispanic	29 (9.9)	56 (11.5)
Hispanic	235 (80.2)	383 (78.8)
Multiracial, non-Hispanic	4 (1.4)	7 (1.4)
Other, non-Hispanic	6 (2.0)	10 (2.1)
**Ethnicity** (freq., %) ^a^		
Non-Hispanic/Latino	58 (19.8)	103(21.2)
Hispanic or Latino	235 (80.2)	383 (78.8)
**Education** (freq., %) ^a^		
< High School	71 (24.2)	122 (25.1)
High School	83 (28.3)	146 (30)
Some college/technical school	87 (29.7)	129 (26.5)
4-years of college	34 (11.6)	57 (11.7)
Some graduate training after college	18 (6.1)	32 (6.6)
**Maternal Ethnicity by Nativity** (freq., %) ^b^		
Non-Hispanic	58 (19.9)	103 (21.7)
US-Born Hispanic	104 (35.6)	169 (35.6)
Non-US-Born Hispanic	130 (44.5)	203 (42.7)
**Birth Order **^**c**^		
1^st^	100 (34.2)	169 (35.8)
2^nd^	89 (30.5)	141 (29.9)
3^rd^	57 (19.5)	91 (19.3)
4^th^	29 (9.9)	45 (9.5)
5^th^	10 (3.4)	14 (3)
6^th^	7 (2.4)	12 (2.5)

All but 1 participant with first trimester MUF had 3^rd^ trimester MUF

^a^ *N* = 486 for trimester three

^b^ *N* = 292 and *N* = 475 for trimesters one and three respectively

^c^ *N* = 292 and *N* = 472 for trimesters one and three respectively

**Table 2:** Urinary fluoride concentrations by trimester

Fluoride Concentrations	Arithmetic Mean	SD	Median	SE	IQR	5^th^ percentile	95^th^ percentile
**MUF First Trimester** (*N* = 293)	0.72	0.53	0.59	0.03	0.55	0.19	1.67
**MUF Third Trimester** (*N* = 490)	0.75	0.50	0.63	0.02	0.58	0.20	1.78
**MUFsg First Trimester** (*N* = 293)	0.81	0.54	0.65	0.03	0.50	0.28	1.85
**MUFsg Third Trimester** (*N* = 490)	0.92	0.61	0.80	0.03	0.59	0.34	1.89

All but 1 participant with first trimester MUF had 3^rd^ trimester MUF, *MUF* maternal urinary fluoride, *MUFsg* specific-gravity adjusted maternal urinary fluoride, *SD* Standard Deviation, *SE* Standard Error

MUFsg measures were moderately consistent (*N* = 292, ICC = 0.46, 95%C.I. 0.32,0.57) and moderately associated (*N* = 292, ρ = 0.50, *p* < 0.001) between both trimesters. MUF was highly correlated with MUFsg in the first (N = 293, ρ = 0.71, *p* < 0.001) and third (*N* = 490, ρ = 0.74, *p* < 0.001) trimesters.

### Associations of MUFsg with Sociodemographic variables

**Fig. 2:**
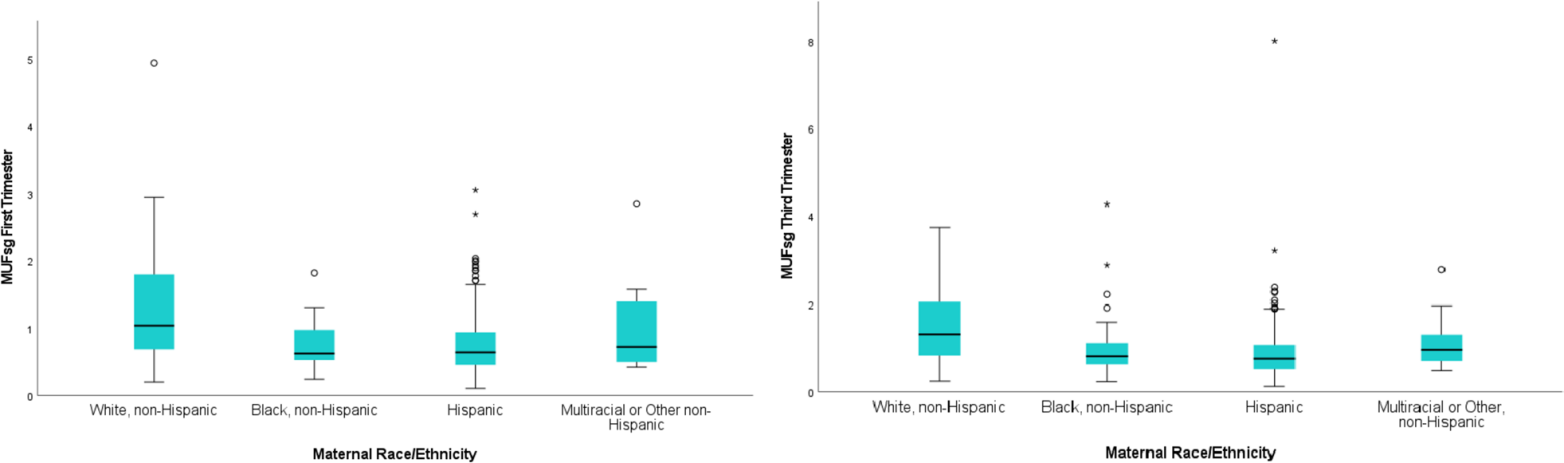
MUFsg concentrations in the first and third trimesters of pregnancy according to race and ethnicity in the MADRES pregnancy cohort MUFsg = specific gravity adjusted maternal urinary fluoride; *N* = 293 for trimester one and *N* = 486 for trimester three

MUFsg levels according to sociodemographic variables at each trimester are presented in Table 3. Maternal age was positively associated with MUFsg during the first (*N* = 293, ρ = 0.16, *p* = 0.006) and third trimesters (*N* = 490, ρ = 0.18, *p* < 0.001), such that older women tended to have higher MUFsg levels. MUFsg also differed by race/ethnicity during the first and third trimesters (*H* (3) = 7.99, *p* = 0.046 and *H* (3) = 25.31, *p* < 0.001 respectively) (see Fig. 2). Specifically, MUFsg was higher for White, non-Hispanic participants than for Hispanic participants in both trimesters *p* = 0.048 and *p* = 0.006 respectively. Additionally, during trimester three, MUFsg was higher for White, non-Hispanic participants than for Black non-Hispanic participants *p* = 0.009. MUFsg differed according to income in both trimesters (*H* (5) = 14.67,* p* = 0.012 and *H* (5) = 29.73, *p* < 0.001 respectively). Specifically, in trimester 1, MUFsg tended to be higher among participants earning $100,000 or more than those earning $15,000 to $29,999 (*p* = 0.03). In trimester 3, MUFsg tended to be higher among participants earning $100,000 or more than those reporting all other income categories (all *p value*s = 0.02). In trimester 3, MUFsg differed according to maternal ethnicity by nativity (*H* (2) = 16.25, *p* < 0.001) and parity (*H (2)* = 9.46, *p* = 0.009). It was higher for non-Hispanic participants than for US-born or non-US-born Hispanic participants (*p*s = 0.003 and 0.002 respectively) and higher for women pregnant with their first child compared to women pregnant with their second child (*p* = 0.021).

**Table 3:** Specific gravity adjusted maternal urinary fluoride concentrations according to select sociodemographic variables

	**First Trimester**						**Third Trimester**					
	**N**	**Median**	**IQR**	**Min**	**Max**	***p***	**N**	**Median**	**IQR**	**Min**	**Max**	***p***
**NIH Race/Ethnicity Categories**												
White, non-Hispanic	19	1.03	1.31	0.20	4.93	*p* = 0.046	30	1.32	1.24	0.25	3.73	*p* < 0.001
Black, non-Hispanic	29	0.62	0.47	0.24	1.81		56	0.82	0.49	0.24	4.27	
Hispanic	235	0.64	0.48	0.11	3.05		383	0.76	0.55	0.13	7.99	
Multiracial or Other, non-Hispanic	10	0.72	0.95	0.42	2.85		17	0.95	0.65	0.48	2.79	
**Education**												
< High School	71	0.68	0.50	0.17	3.05	*p* = 0.031	122	0.73	0.55	0.25	3.21	*p* = 0.050
High School	83	0.62	0.43	0.18	1.98		146	0.80	0.53	0.19	4.27	
Some college/technical school	87	0.61	0.51	0.11	4.93		129	0.76	0.56	0.13	7.99	
4-years of college	34	0.74	0.51	0.20	2.03		57	0.81	0.85	0.18	2.89	
Some graduate training after college	18	1.02	1.09	0.31	2.94		32	0.97	0.86	0.29	3.73	
**Income**												
Don’t know	78	0.70	0.52	0.10	4.93	*p* = 0.012	143	.76	.65	.13	2.89	*p* < 0.001
Less than $15,000	66	0.63	0.47	0.26	3.05		101	.75	.46	.26	4.27	
$15,000 to $29,999	70	0.54	0.31	0.17	2.85		128	.75	.75	.25	7.99	
$30,000 to $49,999	44	0.74	0.56	0.31	1.89		58	.91	.91	.29	2.27	
$50,000 to $99,999	16	0.84	0.62	0.41	2.94		27	.80	.48	.38	2.15	
$100,000 or more	19	0.93	0.93	0.38	2.00		29	.80	.78	.53	3.73	
**Maternal Ethnicity by Nativity**												
Non-Hispanic	58	.76	.59	.20	4.93	*p* = 0.095	103	.94	.76	.24	4.27	*p* < .001
US-Born Hispanic	104	.67	.44	.18	2.69		169	.74	.54	.22	7.99	
Non-US Born Hispanic	130	.61	.51	.10	3.05		203	.79	.62	.13	3.21	
**Birth Order**												
First Born	100	.71	.58	.20	4.93	*p* = 0.190	169	.84	.70	.25	7.99	*p* = 0.009
Second Born	89	.62	.51	.10	2.32		141	.74	.59	.13	4.27	
Third Born or greater	103	.64	.50	.27	3.05		162	.76	.55	.19	2.39

In trimester one, MUFsg also differed by education (*H* (4) = 10.61, *p* = 0.031), in that it was higher for participants with some graduate training than those with high school or some college/technical school education (*p*s = 0.03 and 0.04, respectively). MUFsg was not associated with pre-pregnancy BMI in either trimester (N = 293, ρ = 0.02, *p* = 0.71; N = 490, ρ = 0.03, *p* = 0.54, respectively) trimester, nor did MUFsg differ according to BMI category in either trimester (*H* (3) = 2.6, *p* = 0.46, and *H* (3) = 0.93, *p* = 0.82 respectively). There were also no differences in MUFsg according to smoking history in either trimester (*p* = 0.63 and *p* = 0.51 respectively.

### Associations of MUFsg with blood and urinary metals

Associations between MUFsg and blood and urinary metals are presented in Supplemental Tables S2-S5. MUFsg was negatively associated with blood mercury within trimester one (B = -0.132, 95% CI: -0.233, -0.030, *p* = 0.044) and positively associated with blood lead within trimester three (B = 0.194, 95% CI: 0.076, 0.311, *p* = 0.008). Within both trimesters one and three, MUFsg was positively associated with urinary antimony, barium, cobalt, copper, lead, nickel, and tin(*p*s = 0.008—0.049). Additionally, within trimester one, MUFsg was positively associated with urinary zinc (B = 0.106, 95% CI: 0.042, 0.170, *p* = 0.008), and within trimester three MUFsg was positively associated with urinary cadmium (B = 0.085, 95% CI: 0.026, 0.144, *p* = 0.025). MUFsg during the first trimester was also positively associated with third trimester urinary cadmium, cobalt, and tin (all *p*s = 0.045). No other associations of MUFsg with blood or urinary metals within or between trimesters were significant after FDR correction, although some were marginally significant. Results did not change appreciably for sensitivity analyses that included only participants who fasted during trimester 3 (See Supplemental Tables S6 and S7).

## Discussion

This study characterized urinary fluoride levels during the first and third trimesters of pregnancy among a cohort of predominately Hispanic pregnant women residing in urban LA. MUFsg levels observed in MADRES (trimester 1 mean = 0.81 mg/L; trimester 3 mean = 0.92 mg/L) were higher than those reported in one other US study, but comparable to those observed among pregnant women in Mexico and Canada for which higher levels have been associated with poorer neurodevelopmental outcomes [13, 33–35]. Specifically, Abduweli et al. (2020) found that among 138 women in Northern and Central California, mean MUFsg levels during the second trimester of pregnancy were 0.63 mg/L [21]. Conversely, studies of pregnant women from Mexico [36] and fluoridated communities in Canada [18] observed mean creatinine-adjusted maternal urinary fluoride and MUFsg levels of 0.87 mg/L and 0.88 mg/L during trimester 3 respectively. Notably, in these studies, higher maternal urinary fluoride levels during pregnancy were associated with higher symptoms of ADHD and/or lower child IQ [33–35]. Interestingly, Hispanic women in MADRES tended to have comparable third trimester MUFsg levels (median = 0.76 mg/L) to women in Mexico, while White non-Hispanic women in MADRES had higher levels (median = 1.32 mg/L). MUFsg may have been lower among pregnant women in the Abduweli et al. (2020) study in Northern/Central California because these women were from regions with low water fluoride levels (i.e., < 0.3 mg/L) as well as levels at or above the recommended concentration of 0.7 mg/L [5]. We also observed increases in MUFsg between trimesters one and three. These findings are consistent with studies conducted in Mexico, Canada, and Poland [18, 20, 37]. It has been suggested that increases in urine fluoride across pregnancy might be attributed to higher fluoride uptake by fetal bone during the first trimester of pregnancy than during the third trimester [18]. Lastly, MUFsg and MUF levels in MADRES were highly correlated, which is also consistent with the moderate to high correlations between MUF and MUFsg observed by Abduweli et al. (2020) [21] and Till et al. (2018) [18].

When examining associations of MUFsg with sociodemographic variables, we observed higher MUFsg levels among pregnant women who were older. These findings are consistent with Till et al. (2018) who observed weak positive correlations between urine fluoride and age among pregnant women in the Canadian-based MIREC cohort [18]. Higher urinary fluoride levels among pregnant women who were older may be due to the accumulation of fluoride in bone over time which is then excreted from the body upon urination [38]. Also consistent with Till et al. (2018), we observed higher MUFsg levels among women with higher levels of educational attainment but did not observe associations of MUFsg with pre-pregnancy BMI or smoking history. However, unlike Till et al. (2018) we found higher MUFsg levels among women who were pregnant with their first child compared to their second child. These findings suggest that there may be greater mobilization of fluoride from bone during the first pregnancy compared to subsequent pregnancies.

White non-Hispanic participants in MADRES tended to have higher MUFsg levels than Black non-Hispanic participants or Hispanic participants; however, the sample size among White non-Hispanic participants was relatively small. Interestingly, other studies have shown that tap water consumption tends to be lower, and bottled water (which tends to be lower in fluoride) consumption higher, among Hispanic and non-Hispanic Black adults, including pregnant women, in the US in comparison to non-Hispanic White adults [39–41]. Furthermore, tap water mistrust in Los Angeles tends to be among the highest in the country when compared to other cities, particularly among Hispanic individuals [42]. A tendency for Hispanic adults to mistrust and consume less tap water may stem partly from negative perceptions about the safety of tap water, due to tap water quality issues in their country of origin [43]. However, there are also current racial/ethnic disparities in exposure to environmental toxicants in tap water that may contribute to increased caution around tap water consumption among minoritized populations [44, 45]. Moreover, Black Americans have been shown to report higher levels of mistrust regarding healthcare and public health interventions in general [46–48]. Therefore, it is possible that lower tap water consumption may have contributed to lower urine fluoride levels among non-Hispanic Black and Hispanic women in MADRES. Future research is needed to examine whether potential fluoride exposure disparities observed in MADRES are generalizable to the overall Los Angeles area and greater US population, as well as to assess beverage consumption patterns, and perspectives on fluoridation according to race/ethnicity among pregnant women living in the US.

Women in MADRES with higher levels of MUFsg during trimester three also tended to have higher levels of blood lead, as well as higher levels of various urinary metals, including antimony, barium, cadmium, copper, cobalt, lead, nickel, and tin. Interestingly, research shows that fluoridation chemicals such as sodium fluoride and hydrofluorosilicic acid can be contaminated with metals, including lead, arsenic, barium and aluminum at varying concentrations [22]. Furthermore, children residing in fluoridated communities within the US have been shown to have higher blood lead levels than children residing in communities not treated with fluoridation chemicals [49]. The corrosion of lead-bearing plumbing by fluoridation chemicals administered along with disinfecting agents such as chloramine is one possible pathway by which fluoride and lead co-exposure may occur [50]. However, animal research has also shown that fluoride increases absorption of lead in blood and calcified tissues, potentially by impacting intestinal absorption and/or renal excretion of lead [51]. Therefore, higher blood lead in relation to fluoride exposure among pregnant women in the US may result from co-occurring fluoride and lead exposure in drinking water or increased bodily uptake of lead from other sources by fluoride.

In terms of other metals, fluoride and cadmium can naturally co-occur in drinking water due to industrial processes, thus increasing the likelihood of simultaneous exposure [52]. Interestingly, animal studies point to potential interactions between fluoride and cadmium in affecting renal and hepatic function [23]; however, effects on other health outcomes have yet to be examined. Prior research has also shown that blood copper levels tend to be lower among both adults [53] and children [54] with chronic fluorosis. Therefore, higher urinary copper and urinary fluoride levels among pregnant women in MADRES may reflect increased excretion of copper in relation to higher fluoride exposure. Interestingly, women with higher urinary fluoride during trimester one also tended to have lower blood mercury. Future research is needed to explore potential mechanisms underlying this novel finding.

This study has several strengths, including its large sample size, individual measures of exposure assessment, prospective design, measurement of urinary fluoride at two time points, and breadth of sociodemographic variables and metals measured. However, it also has some limitations. First, although women were asked to fast prior to urine and blood collection, not all were able to comply, and of those who did comply, the duration of fasting was not standardized which may have introduced random error. Still, associations between MUFsg and metals in trimester three did not change appreciably when examined only among women who fasted for at least 8 hours. Second, urine collection time was not standardized and only a single urine sample was obtained per participant. Therefore, urinary fluoride measures in this study tended not to assess cumulative fluoride exposure and they may have been influenced by daily behaviors. Third, data on water consumption habits (i.e., consumption of tap versus bottled water) were not available for most participants in this study at the time of writing and therefore were not included. We also do not have measures of total fluoride intake and exposure. Therefore, future research is needed to examine relative contributions of different sources of fluoride exposure to urinary fluoride levels.

## Conclusion

Urinary fluoride levels among pregnant women in Los Angeles are comparable to those observed among pregnant women in Mexico and fluoridated communities in Canada that have been associated with poorer neurodevelopmental outcomes. Consistent with other studies, urinary fluoride levels among women in this study tended to increase across pregnancy. Lower urinary fluoride levels among Hispanic and non-Hispanic Black participants in MADRES compared to non-Hispanic White participants may reflect lower tap water consumption or lower fluoride exposure from other sources. Studies examining whether maternal urinary fluoride levels are associated with neurodevelopmental outcomes in the US are warranted.

### Supplementary Information

**Additional file 1:** **Table S1.** Maternal Demographics According to Fluoride Sample. **Table S2.** Associations of First Trimester MUFsg with Blood Metals according to Trimester. **Table S3.** Associations of Third Trimester MUFsg with Blood Metals according to Trimester. **Table S4.** Associations of First Trimester MUFsg with Urine Metals According to Trimester. **Table S5.** Associations of Third Trimester MUFsg with Urine Metals according to Trimester. **Table S6.** Associations of Third Trimester MUFsg with Third Trimester Blood Metals among Women who Fasted for at Least 8 Hours. **Table S7.** Associations of Third Trimester MUFsg with Third Trimester Urine Metals among Women who Fasted for at Least 8 Hours.

**Additional file 2.**

**Additional file 3:** **Appendix A.** False Discovery Rate (FDR) Correction Methods.

## Data Availability

The datasets used and/or analyzed during the current study are available from the corresponding author on reasonable request.

## Abbreviations

US: United States
LA: Los Angeles
MADRES: Maternal and Developmental Risks from Environmental and Social Stressors
BMI: Body mass index
MUFsg: Specific gravity adjusted maternal urinary fluoride
MUF: Maternal urinary fluoride
CHEAR: Children’s Health Exposure Analysis Resource
Sb: Antimony
As: Arsenic
Ba: Barium
Be: Beryllium
Cd: Cadmium
Cs: Cesium
Co: Cobalt
Cu: Copper
Cr: Chromium
Hg: Mercury
Mn: Manganese
Mo: Molybdenum
Ni: Nickel
Pb: Lead
Pt: Platinum
Sn: Tin
Tl: Thallium
W: Tungsten
U: Uranium
V: Vanadium
Zn: Zinc
LLOD: Lower limit of detection
ICP-MS: Inductively coupled plasma mass spectrometry
QBIC: Quantitative Bio-element Imaging Center
FDR: False discovery rate
SD: Standard deviation
SE: Standard error
IQR: Interquartile range
