# Supplementary material for: Urinary fluoride levels and metal co-exposures among pregnant women in Los Angeles, California
Source: Environ Health. 2023 Oct 26;22:74. doi: 10.1186/s12940-023-01026-2 (PMC10601173; doi:10.1186/s12940-023-01026-2)
Supplement: Supplementary file 1 — Additional file 1: Table S1. Maternal Demographics According to Fluoride Sample. Table S2. Associations of First Trimester MUFsg with Blood Metals according to Trimester. Table S3. Associations of Third Trimester MUFsg with Blood Metals according to Trimester. Table S4. Associations of First Trimester MUFsg with Urine Metals According to Trimester. Table S5. Associations of Third Trimester MUFsg with Urine Metals according to Trimester. Table S6. Associations of Third Trimester MUFsg with Third Trimester Blood Metals among Women who Fasted for at Least 8 Hours. Table S7. Associations of Third Trimester MUFsg with Third Trimester Urine Metals among Women who Fasted for at Least 8 Hours. [file 12940_2023_1026_MOESM1_ESM.docx]

**Table S1: Maternal Demographics According to Fluoride Sample**

|  | **Sample with Urine Fluoride (N=491)** | **Overall MADRES Cohort**  **(N= 966)** |
| --- | --- | --- |
| **Age at Consent** (yrs; M, SE) | 28.86 (0.27) | 28.38 (0.197) |
| **Pre-pregnancy BMI** (freq., %) ^a^  Underweight  Normal  Overweight  Obese | 13 (2.6)  147 (29.9)  154 (31.4)  177 (36) | 19 (2.2)  258 (29.7)  273 (31.4)  319 (36.7) |
| **Maternal Race/Ethnicity** (freq., %) ^b^  White, non-Hispanic  Black, non-Hispanic  Hispanic  Multiracial, non-Hispanic or  Other, non-Hispanic | 30 (6.2)  56 (11.5)  384 (78.9)  17 (3.5) | 46 (5.0)  110 (12.0)  726 (79.3)  33 (3.6) |
| **Maternal Ethnicity** (freq., %) ^b^  Non-Hispanic/Latino  Hispanic or Latino | 103 (21.1)  384 (78.9) | 189 (20.7)  726 (79.3) |
| **Maternal Education** (freq., %) ^b^  < High School  High School  Some college/technical school  4-years of college  Some graduate training after college | 122 (25.1)  146 (30)  130 (26.7)  57 (11.7)  32 (6.6) | 227 (25.5)  293 (32.9)  233 (26.2)  92 (10.3)  46 (5.2) |
| **Maternal Ethnicity by Nativity** (freq., %) ^c^  Non-Hispanic  US-Born Hispanic  Non-US-Born Hispanic | 103 (21.6)  170 (35.7)  203 (42.6) | 189 (24)  276 (35.1)  322 (40.9) |
| **Birth Order** ^d^  1^st^  2^nd^  3^rd^  4^th^  5^th^  6^th^ | 169 (35.7)  142 (30)  91 (19.2)  45 (9.5)  14 (3)  12 (2.5) | 287 (38.3)  235 (31.3)  131 (17.5)  60 (8.0)  21 (2.8)  16 (2.1) |

a. n=869 for pre-pregnancy BMI for the overall MADRES cohort; b. n=487 for race/ethnicity, ethnicity and education for the urine fluoride sample and n=915 for race/ethnicity and ethnicity for the overall MADRES cohort; n=891 for education for the overall MADRES cohort; c. for maternal ethnicity by nativity, n=476 for the urine fluoride sample and n=787 for the overall MADRES cohort; d. for birth order n=473 for the urine fluoride sample and n=750 for the overall MADRES cohort.

**Table S2: Associations of First Trimester MUFsg with Blood Metals according to Trimester**

| **Trimester 1** (n = 123) | | | | | **Trimester 3** (n = 80) | | | |
| --- | --- | --- | --- | --- | --- | --- | --- | --- |
| **Blood Metal** | **B** | **95% CI** | **Uncorrected p Value** | **FDR Corrected *p* Values** | **B** | **95% CI** | **Uncorrected p Value** | **FDR Corrected *p* values** |
| Arsenic | .002 | -.072, .077 | .953 | 0.953 | -.072 | -.170, .027 | .150 | 0.6 |
| Cadmium | -.030 | -.104, .045 | .427 | 0.854 | .044 | -.042, .131 | .313 | 0.626 |
| Lead | -.016 | -.101, .096 | .710 | 0.947 | .031 | -.078, .139 | .572 | 0.572 |
| Mercury | -.132 | -.233, -.030 | **.011*** | **0.044*** | .047 | -.069, .163 | .422 | 0.563 |

*Note.* Adjusted for pre-pregnancy BMI, maternal age, maternal acculturation, income, and parity; a logarithm base 10 transformation was applied to blood metals to satisfy linear regression assumptions; MUFsg = specific-gravity-adjusted maternal urinary fluoride

**Table S3: Associations of Third Trimester MUFsg with Blood Metals according to Trimester**

| **Trimester 1**(n=127) | | | | | **Trimester 3** (n = 90) | | | |
| --- | --- | --- | --- | --- | --- | --- | --- | --- |
| **Blood Metal** | **B** | **95% CI** | **Uncorrected p Value** | **FDR Corrected *p* Values** | **B** | **95%**  **CI** | **Uncorrected p Value** | **FDR Corrected *p* Values** |
| Arsenic | .036 | -.035, .108 | .314 | 0.628 | -.038 | -.154, 0.078 | .513 | .684 |
| Cadmium | -.011 | -0.083, .061 | .759 | 0.759 | .105 | .011, .199 | **.030*** | .06 |
| Lead | .016 | -.067, 0.099 | .706 | 0.941 | .194 | .076, .311 | **.002**** | **.008*** |
| Mercury | -.099 | -.202, .004 | .059 | 0.236 | .019 | -.113, .152 | .772 | .772 |

*Note.* Adjusted for pre-pregnancy BMI, maternal acculturation, income, maternal age, and parity; a logarithm base 10 transformation was applied to blood metals to satisfy linear regression assumptions; MUFsg of 7.99 was removed for these analyses; MUFsg = specific-gravity-adjusted maternal urinary fluoride

**Table S4: Associations of First Trimester MUFsg with Urine Metals According to Trimester**

| **Trimester 1** (n = 291) | | | | | **Trimester 3** (n = 160) | | | |
| --- | --- | --- | --- | --- | --- | --- | --- | --- |
| **Urine Metal** | **B** | **95% CI** | **Uncorrected *p* Value** | **FDR Corrected *p* value** | **B** | **95%CI** | **Uncorrected *p* Value** | **FDR Corrected *p* value** |
| Antimony (Sb) | .072 | .010, .135 | **.023*** | **0.049*** | .073 | .008, .137 | **.028*** | 0.060 |
| Arsenic (As) | -.013 | -.098, .073 | .772 | 0.891 | .033 | -.067, .133 | .511 | 0.548 |
| Barium (Ba) | .231 | .123, .338 | **<.001***** | **0.015*** | .103 | -.040, .246 | .156 | 0.260 |
| Cadmium (Cd) | .028 | -.043, .100 | .434 | 0.592 | .104 | .030, .177 | **.006**** | **0.045*** |
| Caesium (Cs) | .050 | .007, .093 | **.022**** | 0.055 | .064 | .010, .119 | **.020*** | 0.060 |
| Cobalt (Co) | .081 | .021, .141 | **.009*** | 0.027* | .105 | .031, .180 | **.006*** | **0.045*** |
| Copper (Cu) | .080 | .033, .128 | **<.001***** | **0.015*** | .074 | .014, .133 | .015* | 0.075 |
| Lead (Pb) | .204 | .069, .339 | **.003**** | **0.011*** | .121 | -.054, .296 | .174 | 0.261 |
| Manganese (Mn) | .094 | .009, .178 | **.03*** | 0.056 | .033 | -.025, .090 | .262 | 0.302 |
| Mercury (Hg) | .099 | -.010, .207 | .075 | 0.125 | .072 | -.038, .183 | .200 | 0.273 |
| Molybdenum (Mo) | -.015 | -.084, .054 | .673 | 0.841 | .051 | -.029, .131 | .211 | 0.264 |
| Nickel (Ni) | .134 | .074, .193 | **<.001**** | **0.015*** | .083 | .012, .155 | **.023*** | 0.058 |
| Tin (Sn) | .159 | .058, .260 | **.002**** | **0.010*** | .205 | .071, .339 | **.003**** | **0.045*** |
| Titanium (Ti) | .050 | -.037, .137 | .257 | 0.386 | .114 | .020, .207 | **.017**** | 0.064 |
| Zinc (Zn) | .106 | .042, .170 | **.001**** | **0.008**** | .081 | -.007, .169 | .070 | 0.118 |

*Note.* Adjusted for pre-pregnancy BMI, maternal acculturation, maternal age, income, and parity; a logarithm base 10 transformation was applied to urine metals to satisfy linear regression assumptions; participant with MUFsg of 7.99 removed for analyses of three urine metals; MUFsg = specific-gravity-adjusted maternal urinary fluoride

| **Trimester 1** (n = 294) | | | | | **Trimester 3** (n = 278) | | | |
| --- | --- | --- | --- | --- | --- | --- | --- | --- |
| **Urine Metal** | **B** | **95% CI** | **Uncorrected *p* Value** | **FDR Corrected *p* value** | **B** | **95%CI** | **Uncorrected *p* Value** | **FDR Corrected *p* value** |
| Antimony (Sb) | .037 | -.026, .100 | .252 | 0.540 | .086 | .032, .140 | **.002*** | **0.015*** |
| Arsenic (As) | -.078 | -.162, .007 | .070 | 0.350 | .079 | .005, .152 | **.036*** | 0.077 |
| Barium (Ba) | .136 | .023, .250 | **.019*** | 0.285 | .158 | .049, .267 | **.005*** | **0.025*** |
| Cadmium (Cd) | -.024 | -.095, .048 | .511 | 0.767 | .085 | .026, .144 | **.005*** | **0.025*** |
| Caesium (Cs) | .013 | -.030, .057 | .555 | 0.640 | .034 | -.004, .071 | .082 | 0.137 |
| Cobalt (Co) | .024 | -.037, .085 | .435 | 0.725 | .101 | .048, .154 | **<.001**** | **0.015*** |
| Copper (Cu) | .016 | -.032, .064 | .521 | 0.710 | .067 | .021, .112 | **.005*** | **0.025*** |
| Lead (Pb) | .042 | -.096, .179 | .552 | 0.690 | .179 | .040, .319 | **.012*** | **0.036*** |
| Manganese (Mn) | .043^ | -.042, .128 | .324 | 0.608 | .043 | -.051, .137 | .368 | 0.502 |
| Mercury (Hg) | .078 | -.031, .187 | .160 | 0.400 | .012 | -.102, .126 | .838 | >.99 |
| Molybdenum (Mo) | -.059 | -.128, .010 | .091 | 0.341 | .052 | -.004, .108 | .067 | 0.126 |
| Nickel (Ni) | .057 | -.004, .119 | .068 | 0.510 | .108 | .051, .166 | **<.001**** | **0.015*** |
| Tin (Sn) | -.001 | -.105, .103 | .984 | 0.984 | .139 | .035, .243 | **.009**** | **0.034*** |
| Titanium (Ti) | .069 | -.018, .157 | .120 | 0.360 | .046 | -.033, .125 | .255 | 0.383 |
| Zinc (Zn) | .013 | -.053, .078 | .705 | 0.755 | .080 | .006, .153 | **.034*** | 0.085 |

**Table S5: Associations of Third Trimester MUFsg with Urine Metals according to Trimester**

*Note.* Adjusted for maternal age, pre-pregnancy BMI, maternal acculturation, income, and parity; a logarithm base 10 transformation was applied to urine metals to satisfy linear regression assumptions; participant with Trimester 3 MUFsg=7.99 mg/L removed; MUFsg = specific-gravity-adjusted maternal urinary fluoride

**Table S6: Associations of Third Trimester MUFsg with Third Trimester Blood Metals among Women who Fasted for at Least 8 Hours**

| **Trimester 3** (n = 80) | | | |
| --- | --- | --- | --- |
| **Blood Metal** | **B** | **95%**  **CI** | **Uncorrected p Value** |
| Arsenic | -.032 | -.154, 0.091 | .607 |
| Cadmium | .102 | .006, .199 | **.038*** |
| Lead | .192 | .070, .315 | **.003**** |
| Mercury | -.006 | -.143, .131 | .931 |

*Note.* Adjusted for pre-pregnancy BMI, maternal acculturation, income, maternal age, and parity; a logarithm base 10 transformation was applied to blood metals to satisfy linear regression assumptions; MUFsg of 7.99 was removed for these analyses; MUFsg = specific-gravity-adjusted maternal urinary fluoride

**Table S7: Associations of Third Trimester MUFsg with Third Trimester Urine Metals among Women who Fasted for at Least 8 Hours**

| **Urine Metal**  **(n=233)** | **B** | **95% CI** | **Uncorrected *p* Value** |
| --- | --- | --- | --- |
| Antimony (Sb) | .090 | .032, .149 | **.003*** |
| Arsenic (As) | .089 | .011, .167 | **.026*** |
| Barium (Ba) | .147 | .032, .262 | **.013*** |
| Cadmium (Cd) | .093 | .029, .156 | **.004*** |
| Caesium (Cs) | .031 | -.010, .072 | .135 |
| Cobalt (Co) | .104 | .045, .163 | **<.001**** |
| Copper (Cu) | .053 | .004, .101 | **.033*** |
| Lead (Pb) | .153 | .000, .306 | **.05*** |
| Manganese (Mn) | .021 | -.079, .120 | .686 |
| Mercury (Hg) | -.027 | -.148, .094 | .662 |
| Molybdenum (Mo) | .059 | -.002, .120 | .059 |
| Nickel (Ni) | .108 | .046, .170 | **<.001**** |
| Tin (Sn) | .138 | .026, .249 | **.016**** |
| Titanium (Ti) | .037 | -.050, .123 | .402 |
| Zinc (Zn) | .063 | -.015, .141 | .113 |

*Note.* Adjusted for maternal age, pre-pregnancy BMI, maternal acculturation, income, and parity; a logarithm base 10 transformation was applied to urine metals to satisfy linear regression assumptions; MUFsg=7.99 mg/L removed; MUFsg = specific-gravity-adjusted maternal urinary fluoride
