# Supplementary material for: Urinary fluoride levels and metal co-exposures among pregnant women in Los Angeles, California
Source: Environ Health. 2023 Oct 26;22:74. doi: 10.1186/s12940-023-01026-2 (PMC10601173; doi:10.1186/s12940-023-01026-2)
Supplement: Supplementary file 2 — Additional file 2. [file 12940_2023_1026_MOESM2_ESM.docx]

**Participants who completed a first trimester visit**

N= 487

**Participants in MADRES**

**N**= 1130

**Participants who completed a third trimester visit**

N= 600

**Participants with third trimester urine**

N= 588

**Participants with first trimester urine**

N= 434

**Participants with >3 remaining aliquots of urine**

**Trimester 1:** N=426

**Trimester 3:** N=570

Participants with urine in trimester 1 and 3 or trimester 3 N=494

**Samples discarded during fluoride measurement:**

-n=2 due to insufficient sample volume

-n=2 due to lab technician error

**Participants with urinary fluoride data in Trimester 1**

N= 293

**Participants with urinary fluoride data in Trimester 3**

N=490
