# Supplementary material for: Urinary fluoride levels and metal co-exposures among pregnant women in Los Angeles, California
Source: Environ Health. 2023 Oct 26;22:74. doi: 10.1186/s12940-023-01026-2 (PMC10601173; doi:10.1186/s12940-023-01026-2)
Supplement: Supplementary file 3 — Additional file 3: Appendix A. False Discovery Rate (FDR) Correction Methods. [file 12940_2023_1026_MOESM3_ESM.docx]

**Appendix A:** False Discovery Rate (FDR) Correction Methods

We applied the following formula to adjust for multiple testing based on the Benjamini & Hochberg (1995) method:

**FDR Adjusted *p* value** = unadjusted *p*-value*number of hypotheses tested/rank.

For the current study, we utilized a conservative application of the formula such that *p* values < .001 were designated as =.001 but assigned a higher rank than those that were *p*=.001. Furthermore, we assigned the same rank to tied values.

We reproduced the FDR corrected *p* values for regression analyses of MUFsg and metals in SAS using the SAS PROC MULTTEST statement with the FDR function. This approach also applies linear step-up adjustments to request *p*-values that control the FDR as described by Benjamini and Hochberg (1995). Results were similar and are shown below.

**Associations of First Trimester MUFsg with Blood Metals according to Trimester**

| **Trimester 1** (n = 123) | | | | | **Trimester 3** (n = 80) | | | |
| --- | --- | --- | --- | --- | --- | --- | --- | --- |
| **Blood Metal** | **B** | **95% CI** | **Uncorrected p Value** | **FDR Corrected *p* Values** | **B** | **95% CI** | **Uncorrected p Value** | **FDR Corrected *p* values** |
| Arsenic | .002 | -.072, .077 | .953 | 0.953 | -.072 | -.170, .027 | .150 | 0.563 |
| Cadmium | -.030 | -.104, .045 | .427 | 0.854 | .044 | -.042, .131 | .313 | 0.563 |
| Lead | -.016 | -.101, .096 | .710 | 0.947 | .031 | -.078, .139 | .572 | 0.572 |
| Mercury | -.132 | -.233, -.030 | **.011*** | **0.046*** | .047 | -.069, .163 | .422 | 0.563 |

*Note.* Adjusted for pre-pregnancy BMI, maternal age, maternal acculturation, income, and parity; a logarithm base 10 transformation was applied to blood metals to satisfy linear regression assumptions; MUFsg = specific-gravity-adjusted maternal urinary fluoride

| **Trimester 1**(n=127) | | | | | **Trimester 3** (n = 90) | | | |
| --- | --- | --- | --- | --- | --- | --- | --- | --- |
| **Blood Metal** | **B** | **95% CI** | **Uncorrected p Value** | **FDR Corrected *p* Values** | **B** | **95%**  **CI** | **Uncorrected p Value** | **FDR Corrected *p* Values** |
| Arsenic | .036 | -.035, .108 | .314 | 0.627 | -.038 | -.154, 0.078 | .513 | .684 |
| Cadmium | -.011 | -0.083, .061 | .759 | 0.759 | .105 | .011, .199 | **.030*** | .059 |
| Lead | .016 | -.067, 0.099 | .706 | 0.759 | .194 | .076, .311 | **.002**** | **.006*** |
| Mercury | -.099 | -.202, .004 | .059 | 0.235 | .019 | -.113, .152 | .772 | .772 |

**Associations of Third Trimester MUFsg with Blood Metals according to Trimester**

*Note.* Adjusted for pre-pregnancy BMI, maternal acculturation, income, maternal age and parity; a logarithm base 10 transformation was applied to blood metals to satisfy linear regression assumptions; MUFsg of 7.99 was removed for these analyses; MUFsg = specific-gravity-adjusted maternal urinary fluoride

| **Trimester 1** (n = 291) | | | | | **Trimester 3** (n = 160) | | | |
| --- | --- | --- | --- | --- | --- | --- | --- | --- |
| **Urine Metal** | **B** | **95% CI** | **Uncorrected *p* Value** | **FDR Corrected *p* value** | **B** | **95%CI** | **Uncorrected *p* Value** | **FDR Corrected *p* value** |
| Antimony (Sb) | .072 | .010, .135 | **.023*** | **0.039*** | .073 | .008, .137 | **.028*** | 0.053 |
| Arsenic (As) | -.013 | -.098, .073 | .772 | 0.772 | .033 | -.067, .133 | .511 | 0.511 |
| Barium (Ba) | .231 | .123, .338 | **<.001***** | **0.0002*** | .103 | -.040, .246 | .156 | 0.234 |
| Cadmium (Cd) | .028 | -.043, .100 | .434 | 0.501 | .104 | .030, .177 | **.006**** | **0.030*** |
| Caesium (Cs) | .050 | .007, .093 | **.022**** | 0.0391 | .064 | .010, .119 | **.020*** | **0.049*** |
| Cobalt (Co) | .081 | .021, .141 | **.009*** | 0.019* | .105 | .031, .180 | **.006*** | **0.030*** |
| Copper (Cu) | .080 | .033, .128 | **<.001***** | **0.0043*** | .074 | .014, .133 | .015* | **0.049*** |
| Lead (Pb) | .204 | .069, .339 | **.003**** | **0.008*** | .121 | -.054, .296 | .174 | 0.237 |
| Manganese (Mn) | .094 | .009, .178 | **.03*** | **0.044*** | .033 | -.025, .090 | .262 | 0.280 |
| Mercury (Hg) | .099 | -.010, .207 | .075 | 0.103 | .072 | -.038, .183 | .200 | 0.244 |
| Molybdenum (Mo) | -.015 | -.084, .054 | .673 | 0.721 | .051 | -.029, .131 | .211 | 0.244 |
| Nickel (Ni) | .134 | .074, .193 | **<.001**** | **0.0002*** | .083 | .012, .155 | **.023*** | **0.049*** |
| Tin (Sn) | .159 | .058, .260 | **.002**** | **0.006*** | .205 | .071, .339 | **.003**** | **0.030*** |
| Titanium (Ti) | .050 | -.037, .137 | .257 | 0.321 | .114 | .020, .207 | **.017**** | **0.049*** |
| Zinc (Zn) | .106 | .042, .170 | **.001**** | **0.0048**** | .081 | -.007, .169 | .070 | 0.118 |

**Associations of First Trimester MUFsg with Urine Metals According to Trimester**

*Note.* Adjusted for pre-pregnancy BMI, maternal acculturation, maternal age, income, and parity; a logarithm base 10 transformation was applied to urine metals to satisfy linear regression assumptions; participant with MUFsg of 7.99 removed for analyses of three urine metals; MUFsg = specific-gravity-adjusted maternal urinary fluoride

| **Trimester 1** (n = 294) | | | | | **Trimester 3** (n = 278) | | | |
| --- | --- | --- | --- | --- | --- | --- | --- | --- |
| **Urine Metal** | **B** | **95% CI** | **Uncorrected *p* Value** | **FDR Corrected *p* value** | **B** | **95%CI** | **Uncorrected *p* Value** | **FDR Corrected *p* value** |
| Antimony (Sb) | .037 | -.026, .100 | .252 | 0.541 | .086 | .032, .140 | **.002*** | **0.010*** |
| Arsenic (As) | -.078 | -.162, .007 | .070 | 0.340 | .079 | .005, .152 | **.036*** | 0.053 |
| Barium (Ba) | .136 | .023, .250 | **.019*** | 0.286 | .158 | .049, .267 | **.005*** | **0.012*** |
| Cadmium (Cd) | -.024 | -.095, .048 | .511 | 0.640 | .085 | .026, .144 | **.005*** | **0.012*** |
| Caesium (Cs) | .013 | -.030, .057 | .555 | 0.640 | .034 | -.004, .071 | .082 | 0.103 |
| Cobalt (Co) | .024 | -.037, .085 | .435 | 0.640 | .101 | .048, .154 | **<.001**** | **0.002*** |
| Copper (Cu) | .016 | -.032, .064 | .521 | 0.640 | .067 | .021, .112 | **.005*** | **0.012*** |
| Lead (Pb) | .042 | -.096, .179 | .552 | 0.640 | .179 | .040, .319 | **.012*** | **0.022*** |
| Manganese (Mn) | .043^ | -.042, .128 | .324 | 0.608 | .043 | -.051, .137 | .368 | 0.395 |
| Mercury (Hg) | .078 | -.031, .187 | .160 | 0.399 | .012 | -.102, .126 | .838 | .838 |
| Molybdenum (Mo) | -.059 | -.128, .010 | .091 | 0.340 | .052 | -.004, .108 | .067 | 0.092 |
| Nickel (Ni) | .057 | -.004, .119 | .068 | 0.340 | .108 | .051, .166 | **<.001**** | **0.002*** |
| Tin (Sn) | -.001 | -.105, .103 | .984 | 0.984 | .139 | .035, .243 | **.009**** | **0.020*** |
| Titanium (Ti) | .069 | -.018, .157 | .120 | 0.361 | .046 | -.033, .125 | .255 | 0.294 |
| Zinc (Zn) | .013 | -.053, .078 | .705 | 0.756 | .080 | .006, .153 | **.034*** | 0.053 |

**Associations of Third Trimester MUFsg with Urine Metals according to Trimester**

*Note.* Adjusted for maternal age, pre-pregnancy BMI, maternal acculturation, income, and parity; a logarithm base 10 transformation was applied to urine metals to satisfy linear regression assumptions; participant with Trimester 3 MUFsg=7.99 mg/L removed; MUFsg = specific-gravity-adjusted maternal urinary fluoride

References

Benjamini, Y., & Hochberg, Y. (1995). Controlling the False Discovery Rate: A Practical and Powerful Approach to Multiple Testing. *Journal of the Royal Statistical Society*. Series B (Methodological), 57(1), 289–300. http://www.jstor.org/stable/2346101
